# The immediate effect of traditional Malay massage on substance P, inflammatory mediators, pain scale and functional outcome among patients with low back pain: study protocol of a randomised controlled trial

**DOI:** 10.1186/s12906-016-0988-1

**Published:** 2016-01-15

**Authors:** Nurhanisah Sejari, Kamaria Kamaruddin, Kalavathy Ramasamy, Siong Meng Lim, Chin Fen Neoh, Long Chiau Ming

**Affiliations:** 1 Physiotherapy Department, Faculty of Health Sciences, Universiti Teknologi MARA (UiTM), Puncak Alam, Malaysia; 2 Faculty of Pharmacy, Universiti Teknologi MARA (UiTM), Bandar Puncak Alam, 42300 Selangor Darul Ehsan Malaysia; 3 Collaborative Drug Discovery Research (CDDR) Group, Pharmaceutical and Life Sciences CoRe, Universiti Teknologi MARA (UiTM), Shah Alam, 40450 Selangor Darul Ehsan Malaysia; 4 Brain Degeneration and Therapeutics Group, Pharmaceutical and Life Sciences CoRe, Universiti Teknologi MARA (UiTM), Shah Alam, 40450 Selangor Darul Ehsan Malaysia

**Keywords:** Low back pain, Massage, Substance P, Visual analogue pain scale, East Asian traditional medicine

## Abstract

**Background:**

The treatment of low back pain is very challenging due to the recurrent nature of the problem. It is believed that traditional Malay massage helps to relieve such back pain but there is a lack of scientific evidence to support both the practice of traditional Malay massage and the mechanism by which it exerts its effect. The aim of this study is to investigate the immediate effect of traditional Malay massage on the pain scale, substance P, inflammatory mediators, and functional outcomes among low back pain patients.

**Methods:**

A non-blinded, randomised controlled trial will be conducted. A total of sixty-six patients who fulfil the inclusion criteria will be recruited. The participants will be randomly allocated into intervention (traditional Malay massage) and control (relaxation position) groups. Blood and saliva samples will be collected before and immediately after intervention. All collected samples will be analysed. The primary outcomes are the changes in the level of substance P in both saliva and blood samples between both groups. The secondary outcomes include the levels of inflammatory mediators [i.e. TNF-α, IL-1β, IL-8, monocyte chemotactic protein-1, IL-6 and IL-10, and the soluble form of the intercellular adhesion molecule], the pain intensity as measured by a visual analogous scale and functional outcomes using the Roland-Morris Disability Questionnaire.

**Discussion:**

Massage is a type of physical therapy that has been proven to be potentially capable of reducing unpleasant pain sensations by a complex sensory response and chemical mediators such as substance P and various inflammatory mediators. Previous studies conducted using Thai, Swedish, or other forms of massage therapies, have showed inconsistent findings on substance P levels pre and post the interventions. Each massage genre varies in terms of massage and joint mobilization points, as well as the lumbar spinous process. Traditional Malay massage, known locally as “Urut Melayu”, involves soft-tissue manipulation of the whole body applied using the hands and fingers. This massage technique combines both deep muscular tissue massage and spiritual rituals. This trial is expected to give rise to new knowledge underlying the mechanisms for pain and inflammation relief that are activated by traditional Malay massage.

**Trial registration:**

Australian New Zealand Clinical Trials ACTRN12615000537550.

## Background

It has been reported that approximately 40–60 % Malaysians suffer from low back pain (LBP) [1]. A recent study by Hong et al. in the United Kingdom indicated that the total annual health care costs for patients with back pain were more than double than those of matched controls [2]. Pain is an unpleasant sensation, involving a complex sensory response due to tissue damage. The pain intensity varies from mild pain to severe pain sensations that require treatment. When there is cellular damage in the body, chemical mediators are released and detected by sensory neurons, resulting in the activation of pain receptors. There are many chemical mediators involved in periods of pain and inflammation, including substance P (SP).

SP is a neuropeptide produced in a subset of capsaicin sensitive sensory peripheral neuron cell bodies localised in dorsal root and trigeminal ganglia, which plays a pivotal role in the transmission of noxious stimuli in the spinal cord [3]. Structurally, SP is an 11-amino acid polypeptide whose C-terminal amino acid sequence is essential for its pharmacological activities. It preferentially binds to the neurokinin 1 (NK1) receptor. After binding to this receptor, SP modifies Ca^2+^ and K^+^currents at the cellular level [3]. The role of SP in the onset of pain and inflammation is being increasingly recognised [4, 5]. The physiology involved in the initiation of and response to muscle pain is associated with activation of the group C afferent nerve fibres and is usually accompanied by a certain degree of tissue damage. This in turn results in the release of chemical mediators, including SP, from the damaged cells and the activation of nociceptor nerve endings. These chemical mediators, especially SP, sensitize the nociceptors’ response to normal stimuli by altering the transduction properties of the free nerve endings [6, 7].

The role of SP in the pathophysiology of clinical syndromes such as inflammatory joint disease and diseases with chronic neuropathic and inflammatory pain in general is becoming clearer [8]. There is a growing awareness that improved knowledge of SP may allow the development of drugs and interventions which target the modulation of SP so as to be able to help in treating the pain associated with these diseases. Many studies have used the level of SP in saliva as an indicator of the severity of pain in chronic pain conditions, since the amount of SP is significantly greater in saliva than in plasma. Also, the non-invasive nature of saliva collection suggests that SP in saliva may be useful as an alternative neurochemical correlation with chronic LBP [9–12].

Current analgesic therapy using non-steroidal anti-inflammatory drugs does not treat back pain effectively and, with long term use, is associated with kidney failure [13]. On the other hand, physical therapies, including massage and spinal manipulation, are known for their beneficial effect on pain reduction, and it has been suggested that this may be due to a reduction of the SP level in patients with LBP [12, 14]. The popularity of alternative medical treatment has increased for many conditions in recent years, and massage has been documented as one of the most frequently used alternative treatments for back pain [15].

The traditional Malay massage (TMM), known locally as “Urut Melayu”, involves deep friction and muscular massage of the whole body by the practitioner exclusively using their hands and fingers. It is culturally believed that this TMM healing is partially spiritual in nature [16, 17]. In the therapeutic aspect, TMM provides pain treatment as well as involvement of musculoskeletal problems such as muscle spasm. Other than that, it also helps in psychological aspects such as providing relaxation, improving confidence levels and reducing anxiety [17]. To date, ten government hospitals under the Ministry of Health, Malaysia offer TMM for the management of chronic pain under Traditional and Complementary Medicine units [18]. In 2011, 13,530 patients received TMM treatment in these units [19]. Although TMM is very popular, both in Malaysia but other countries in the Asian region [17], the acute and sustained effect of TMM on the aching muscle and its analgesic and anti-inflammatory mechanism remains unknown.

There is a lack of scientific evidence, however, to support the practice of TMM and the mechanism by which it reduces back pain. It has been postulated that there is a link between the positive effect of TMM and the presence of SP in patients with LBP [12]. The aim of this study, therefore, is to investigate the immediate effect of TMM on SP, inflammatory mediators, pain intensity and quality of life among LBP patients. The results of this research will be instrumental in elucidating the value of TMM in human pathology.

## Methods

### Study design

A non-blinded, 2-arm randomized controlled trial will be conducted. A total of 66 eligible participants will be randomly assigned into two groups: (i) TMM or (ii) control groups, using a simple random sampling method. Random allocation to either the intervention or control group is achieved by sequentially numbered, sealed opaque envelopes containing the group allocation, which will be determined by a computer-generated random number. A research assistant with no direct contact with the participants will be responsible for generating the random numbers and preparing the envelopes. Ethical approval has been obtained from the UiTM Research Ethics Committee [600-RMI(5/1/6)].

### Patient recruitment

LBP patients who fulfil the inclusion criteria, will be screened by a Physiotherapist and Orthopaedic specialist and will be invited to participate in this study. Inclusion criteria will be as follows: has experienced an episode of LBP of more than four weeks’ duration with a score on the visual analogue scale (VAS) of at least 4, is aged between 20 and 40 years old and has a body mass index (BMI) of between 18.5 kg/m^2^ and 29.9 kg/m^2^. The patients will be excluded if they suffer from identified health problems such as nerve involvement or compression, spinal disorders (e.g., spondylolisthesis, herniated nucleus pulposus, spondylosis, spinal fracture, back surgery), neurological deficit (e.g., multiple sclerosis, hemi/ para-paresis or myelopathy), and infectious diseases (e.g., AIDS or tuberculosis). Smokers and those experiencing menstruation, pregnancy or febrile convulsion during the study will also be excluded. LBP patients who are taking medication containing analgesics or steroids, and those who have sought treatment for LBP in the past two weeks will also be excluded.

An explanatory statement will be given to those deemed to be eligible to participate, and written informed consent will be obtained before the commencement of the study. Consenting participants will then be randomly assigned into the two groups as described above.

### Intervention

**Fig. 1:**
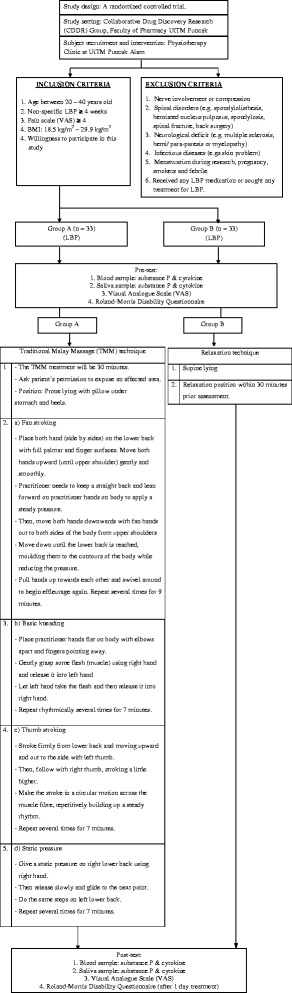
Study protocol

The patients in the TMM group will be treated by one certified practitioner of TMM, with the treatment being applied for 30 min in one session only on the area of pain in the lower back. The practitioner will follow the protocol of TMM that has been provided for and approved by the Ministry of Health, Malaysia [18]. The massage techniques involved in TMM are fan stroking, basic kneading, thumb stroking, and static pressure (Fig. 1). On the other hand, the patients in the control group will undergo a relaxation position for 30 min (Fig. 1).

### Outcome measures

The primary outcome measure for this study is the difference in the level of SP in blood and saliva samples pre- and post-intervention. All samples will be analysed at the Collaborative Drug Discovery Research (CDDR) Laboratory, Faculty of Pharmacy, UiTM Puncak Alam Campus.

The secondary outcome measures include the levels of inflammatory mediators [i.e., TNF-α, IL-1β, IL-8, monocyte chemotactic protein-1 (MCP-1), IL-6 and IL-10, and the soluble form of intercellular adhesion molecule (sICAM-1)] in both blood and saliva samples [20]. Other secondary outcome measures also include the visual analogous scale (VAS) score (indicating the pain intensity) and the Roland-Morris Disability Questionnaire (RMDQ) [21] score (assessing the level of disability associated with LBP).

### Instruments

Both blood and saliva samples will be collected and centrifuged (10,000 g and 4 °C for 15 min) to eliminate debris and cellular matter. Then, the Human Substance P EIA kit (Cusabio Biotech; CSB-E08357h) will be used to determine the level of SP. Other inflammatory mediators, such as TNF-α (eBioscience; BMS2034TEN), IL-1β (Cayman chemical;583311-96 well), IL-6 (BMS213/2TEN), IL-8 (eBioscience; BMS204/3), IL-10 (eBioscience; BMS215/2), monocyte chemotactic protein-1(MCP-1) (eBioscience; BMS281) and the soluble form of the intercellular adhesion molecule (sICAM-1) (Arigo; ARG80215), will be tested individually in both blood and saliva samples using a human Elisa kit (Table 1).

**Table 1:** Analytical and assessment characteristics

No.	Assessment	Explanation
1.	Blood sample	Blood collection
		- 10 ml of venous blood will be taken by a trained nurse.
		- The whole blood will be collected into a plain polyethylene tube until blood clot formation.
		- The clots will be separated from the wall of tube using a wooden applicator stick.
		- The serum will be separated by centrifugation at 10,000 g at 4 °C for 15 min to eliminate debris and cellular matter.
		Blood test
		1) Substance P
		- The blood serum will be tested using Human Substance P EIA kit (Cusabio Biotech)
		2) Cytokines
		- The blood serum will be tested individually using Elisa kits to investigate
		a) TNF-α
		b) IL-1β
		c) IL-6
		d) IL-8
		e) IL-10
		c) monocyte chemotactic protein-1 (MCP-1)
		d) The soluble form of intercellular adhesion molecule (Sicam-1).
2.	Saliva sample	Saliva collection
		- 3 to 4 ml of unstimulated (resting) whole saliva will be collected 2 min after the patient has rinsed his/hermouth several times with tap water.
		- The accumulated saliva in the floor of the mouth will be drawn by a plastic disposable pipette and collected into a plastic polyethylene tube.
		- The collected saliva will be centrifuged at 10,000 g at 4 °C for 15 min to eliminate debris and cellular matter.
		Blood test
		1) Substance P
		- The blood serum will be tested using Human Substance P EIA kit (Cusabio Biotech)
		2) Cytokines
		- The blood serum will be tested individually using Elisa kits to investigate:
		a) TNF-α
		b) IL-1β
		c) IL-6
		d) IL-8
		e) IL-10
		c) monocyte chemotactic protein-1 (MCP-1)
		d) The soluble form of intercellular adhesion molecule (sICAM-1).
3.	Visual Analogue Scale (VAS)	- Pain intensity will be measured using VAS by asking the patients to describe their pain level before and after treatment.
4.	Roland-Morris Disability questionnaire	- Functional disability will be measured using the Roland-Morris Disability questionnaire.

The VAS is a measurement of pain intensity and has been proven to be reliable and valid [22, 23]. The VAS scale ranges from ‘0’ to ‘10’ with a different facial expression whereby 0 represents ‘no pain’ and 10 represents the ‘worst possible pain’. Patients will be asked to rate their pain level using the VAS pre- and post- intervention.

The RMDQ is an instrument for assessing the level of disability associated with LBP [21]. RMDQ contains 24 items which relate to several activities in daily life. The scoring depends on the patient’s ability in doing the daily tasks. The survey will be taken one day before and one day after the intervention.

### Sample size calculation

The sample size is calculated using Power and Sample Size (PS) software 3.1.2 version [24]. The difference between the mean values of the intervention and control groups, and the standard deviation (post-massage) of SP levels were used as parameters for sample size calculation. Since there is no previous finding of the effect of TMM on substance P, we chose the closest related result based on traditional Thai massage on LBP patients reported by MacKawan et al. [12]. Their study reported that the mean levels of SP in the intervention group and control group are 50.43 pg/mL; 56.27 pg/mL, respectively (the difference in the mean values between the experimental and control groups is 5.84 pg/mL). Their findings also showed that the response within each subject group was normally distributed, with the highest recorded standard deviation being 8.3. The Type I error probability associated with this test of the null hypothesis is 0.05. A sample of 33 experimental subjects and 33 control subjects is therefore required to reject the null hypothesis that the population means of the experimental and control groups are equal, with a probability (power) of 0.8.

### Data analysis plan

Data from participants will be entered into a computerised database and analysed using SPSS software version 20.0. The mean, standard deviation and range will be calculated for each variable. Data will be checked for a normal distribution in terms of skewness and kurtosis, normality test, univariate outliers and missing data. Data will be presented in table and line graphs. The paired *t*-test will be used to measure the differences pre- and post-intervention within and between groups for every outcome measure. A *p*-value of less than 0.05 will be used to show that the result is statistically significant.

## Discussion

This study is designed to investigate how SP and inflammatory mediators might contribute to the pain reduction and inflammation relief apparently offered by TMM. Generally, therapeutic massage involves the manipulation of the soft tissue of the whole body area in a way that contributes to health improvements, including relaxation, improved sleep and specific physical benefits, such as relief of muscular aches and pain [25]. Gentle stretching of the joints and muscles relieves tension, enhances flexibility, and induces a deep state of tranquillity [26]. TMM appears to be different from other forms of massage or physical manipulations: it is performed using systematic manipulation of the soft tissue of the body for pain reduction or other therapeutic purposes. Basically, it is a deep massage with prolonged pressure on the muscles along with passive stretching. Pressure point massage alone is believed to release blocked energy, and to increase awareness and vitality [27, 28]. TMM practitioners describe that this massage can optimise blood flow and relieve the patient of wind (local term: *angin*), which if left untreated can turn into mucus (local term: *lendir*) and finally solid lumps (local term: *ketulan*). These effects could relieve and control back pain [27, 28].

This is the first ever study conducted to quantify the pain and inflammatory mediators present before and after TMM. It must be noted, however, that MacKawan et al. [12] have previously investigated the effects of traditional Thai massage on SP and pain perception in patients with non-specific LBP. This type of Thai massage resembles TMM as it also emphasises deep friction using repetitive hands movement. Similar to our trial, their study was a randomized controlled trial in which the volunteers in the interventional arm were given 10 min of Thai massage. Their findings reported a significant decrease in the SP level in saliva after the Thai massage treatment [12]. This outcome was supported by Fields et al. who revealed that Swedish massage (a type of superficial massage) reduced the SP level [14]. Furthermore, the VAS pain score in the Thai massage group showed the presence of less pain than in the control group (Mean VAS score of 2.48 in the massage versus 3.36 in the control group). Meanwhile, another randomized controlled trial investigating the immediate effects of spinal manipulation on SP and pain perception on asymptomatic volunteers has been reported [29]. A total of 30 subjects were randomly assigned equally into three groups (control, cervical spine and dorsal spine manipulation). Unlike the study conducted using Thai massage [12], significant increases in SP levels immediately and 2 h after treatment were reported in the cervical manipulation groups. Furthermore, the pain pressure threshold level was elevated immediately after the spinal manipulation compared to the control group. Based on the results of their study, Molina-Ortega et al. postulated that the pain threshold level is increased when the SP increases. The caveats in these previous studies were that healthy volunteers were used to investigate the changes of SP post intervention and the control groups were exposure to certain degrees of manipulation and massage which could inadvertently have caused physiological changes.

In Malaysia, TMM is one of the most popular alternative treatments. Thus, the present project, will be the first to provide new knowledge on the immediate effects of TMM on pain and inflammation relief, in the context of investigating the underlying mechanism of relief by reductions in SP. In addition, the control group in this study will be given a relaxation technique rather than a treatment that might show the same effect as massage therapy. Furthermore, other inflammatory mediators will also be measured to show their involvement in the pain and inflammation process among LBP patients. The difference in the response towards treatment among the patients could also lead to genomic related studies investigating the effect of SP in terms of human genetic. Since the current scientific evidence is limited, this experimental study can provide the necessary evidence to prescribe TMM as an alternative or additional complementary treatment for LBP.
